# Mitigating hemoglobin‐induced nephropathy: ApoHb‐hp protection of podocytes

**DOI:** 10.14814/phy2.70132

**Published:** 2024-11-22

**Authors:** Daniela Lucas, Carlos Munoz, Quintin O'Boyle, Ivan S. Pires, Andre F. Palmer, Pedro Cabrales

**Affiliations:** ^1^ Department of Bioengineering University of California San Diego San Diego California USA; ^2^ William G. Lowrie Department of Chemical and Biomolecular Engineering The Ohio State University Columbus Ohio USA

**Keywords:** filtration barrier, Hb scavenging, hemoglobin, heme scavenging, hemoglobin toxicity, podocytes, renal damage, ROS

## Abstract

This study investigates hemoglobin (Hb)‐induced kidney injury and the protective role of the ApoHemoglobin‐Haptoglobin (ApoHb‐Hp) complex against heme and Hb damage. Hb facilitates oxygen (O_2_) delivery but poses challenges outside red blood cells (RBCs) due to toxic Hb and heme mechanisms. These are managed by binding to serum proteins like Haptoglobin (Hp) and Hemopexin (Hpx). During hemolysis, depletion of Hp and Hpx leaves tissues vulnerable to Hb and heme. To address this, we developed the ApoHb‐Hp complex, based on Apohemoglobin, which is produced by removing heme from Hb, conjugated with Hp. This complex acts as a dual scavenger for Hb and heme, preventing tissue damage. Our findings demonstrate that ApoHb‐Hp significantly protects MPC5 podocytes from Hb‐induced damage. Fluorescent staining showed a higher percentage of nephrin‐positive cells in the ApoHb‐Hp group, and MTT assays revealed enhanced cell viability compared to Hb alone. Additionally, ApoHb‐Hp reduced reactive oxygen species (ROS) production, with the Hb group exhibiting significantly elevated ROS levels. The ApoHb‐Hp complex mitigated the depletion of protective mechanisms, as shown by significant increases in superoxide dismutase (SOD) and glutathione (GSH). Moreover, ApoHb‐Hp treatment reduced the activation of the NLRP3 inflammasome signaling pathway and inflammatory cytokines IL‐1β and IL‐18. These findings underscore the therapeutic potential of ApoHb‐Hp in mitigating Hb‐induced renal damage by preserving podocyte viability and reducing oxidative stress. Overall, ApoHb‐Hp maintained protective mechanisms depleted otherwise by Hb. These findings highlight ApoHb‐Hp's potential as a therapeutic agent against Hb‐induced renal damage, offering insights into its mechanisms and implications for treating conditions involving hemolysis.

**New and Noteworthy**

This research elucidates the diverse mechanisms through which Hb toxicity leads to renal injury. Furthermore, it highlights the protective capacity of ApoHb‐Hp against Hb‐induced renal damage. By delving into the disturbance of filtration barrier cells, particularly podocytes, we gain insight into the potential systemic repercussions on renal function. This underscores the intricate interplay between Hb toxicity and renal pathology, emphasizing the significance of interventions like ApoHb‐Hp in preserving renal integrity and function amidst Hb‐related challenges.

## 1 INTRODUCTION

Hemolysis, the rupture of red blood cell membranes leading to the release of hemoglobin (Hb), is a physiological process managed by intrinsic proteins like haptoglobin (Hp) and hemopexin (Hpx) in mammalian organisms (Buehler et al., 2020; Deuel et al., 2015; Tolosano & Altruda, 2002). These proteins are crucial in mitigating the potential adverse effects of minor hemolysis, primarily by scavenging circulating Hb and heme released from Hb within the bloodstream (Sears, 1970). However, their limited concentrations in plasma render them insufficient in moderated and severe hemolysis, leading to Hb and heme toxicity and extravasation, generation of reactive oxygen species (ROS), and consequential cellular and tissue damage (Buehler & D'Agnillo, 2010; Deuel et al., 2016; Feola et al., 1988; Muller‐Eberhard et al., 1968; Vallelian et al., 2022). Severe hemolysis can occur in various clinical scenarios, including hemolytic diseases, dialysis, extracorporeal circulation machines, and chemotherapy (Leger et al., 2015; Materne et al., 2021; Sam et al., 2023). Addressing the substantial cases of hemolysis is critical to prevent co‐morbidities associated with these medical conditions and procedures.

The toxicity of cell‐free Hb is multifaceted. First, Hb readily decomposes resulting in the dissociation of the tetramer into αβ‐chain heterodimers (32 kD) or even into smaller monomers (16 kDa) (Huang et al., 2013). These sub‐parts are sufficiently small to extravasate and infiltrate tissues triggering an immune response.  Second, Hb engages with ligands beyond oxygen, particularly nitric oxide (NO) and peroxides, leading to NO depletion, vascular dysfunction, and potential oxidative tissue damage. Third, Ferric Hb (Fe3+), produced through Hb autoxidation or reactions with endogenous oxidants, can release heme. Free heme is a potent trigger of lipid peroxidation and inflammation and acts as an endogenous proteasome inhibitor, compromising cellular integrity and triggering ROS formation. The administration of Hp normalizes vascular NO signaling following hemolysis, limiting extravasation and vasoconstriction by binding with Hb and significantly increasing the size of the resulting Hb‐Hp complex (Schaer et al., 2016). Supplementation of Hp also helps in reducing Hb toxicity by scavenging heme and reducing inflammation and tissue injury (Buehler et al., 2020; Gentinetta et al., 2022).

In the context of renal health, the kidneys are highly vulnerable to hemolysis and cell‐free Hb in circulation in general. Once natural scavenging systems are saturated, the kidneys serve as the primary route for Hb clearance, leading to excessive filtration of cell‐free Hb and heme. Dimeric Hb, when plasma Hp is saturated, can be filtered by the glomerulus and taken up by proximal tubule cells, releasing heme that may contribute to cellular damage and oxidative stress (Dutra et al., 2014). Kidney injury is a common and serious complication of hemolysis, significantly contributing to  morbidity and mortality. The damage to the kidneys from Hb is thought to occur through various mechanisms, such as oxidative stress and cytotoxic pathways, the formation of intratubular casts, and direct or indirect inflammatory responses, particularly through the activation of neutrophils and monocytes. A deeper understanding of the complex pathophysiology behind kidney injury caused by hemolysis could lead to the development and application of new treatments aimed at mitigating the harmful and often lethal impacts of hemolysis on kidney function.

In response to this ongoing challenge, our research group has developed a novel dual scavenger protein named ApoHemoglobin‐Haptoglobin (ApoHb‐Hp) complex. This engineered protein complex binds to free heme and Hb, thereby preventing tissue cytotoxicity (Munoz et al., 2020). To achieve this scavenging of heme and Hb, a hypothesized mechanism of action has been previously described (Munoz et al., 2020). ApoHb, due to its heme vacancy, scavenges free heme, while Hp binds Hb dimers, forming a stable complex that prevents extravasation and heme release (Andersen et al., 2012; Buehler et al., 2009). ApoHb‐Hp's interaction with acellular Hb confines the molecules into a stable complex preventing extravasation and heme release (Schaer et al., 2016). Previous studies have demonstrated the efficacy of ApoHb‐Hp in reducing microvascular constriction and lowering levels of inflammatory cytokines in critical organs, highlighting its potential as a therapeutic candidate (Cabrales et al., 2011; Munoz et al., 2020).

To further explore the protective potential of ApoHb‐Hp, we focused on understanding the impact of Hb on kidney cells and exploring how this therapeutic could prevent renal toxic events (Baek et al., 2018; Deuel et al., 2016; Nath et al., 2022). Podocytes, specialized cells enveloping the capillaries of glomeruli and crucial in establishing the kidney's filtration barrier, were chosen as the primary cells of interest (Kawachi et al., 2006; Reiser & Altintas, 2016). Podocytes achieve their filtration barrier function through the intricate structure of their feet processes, allowing the selective passage of small molecules out of the bloodstream (Kocylowski et al., 2022). Consequently, when free heme and Hb circulate in the bloodstream, podocytes become the first cells in contact during their passage through the circulation and kidneys, rendering them more vulnerable to toxicities. In addition, the dimerization of Hb can disrupt this filtration barrier, leading to detrimental effects on the glomerulus and its podocytes.

In the present study, we aim to comprehend the effect of Hb on isolated podocytes and investigate how ApoHb‐Hp may exert a protective mechanism against Hb‐induced damage. Our study focuses explicitly on assessing cell viability and the upregulation of inflammatory pathways when podocytes are cultured with free acellular Hb. Furthermore, we aim to explore the potential of ApoHb‐Hp as a therapeutic agent capable of scavenging Hb and preventing the upregulation of inflammatory signals, thereby enhancing cell viability.

## 2 METHODS

### 2.1 Samples preparation

The ApoHb‐Hp complex is a combination of ApoHb and Hp in a batch. This mixture is generated by determining a volume ratio, represented as mL of Hp per mL of ApoHb. This ratio indicates the quantity of ApoHb required to be mixed with each unit volume of Hp. The complete description and protocol of the solution used in this article is detailed elsewhere (Munoz et al., 2020). The acellular free Hb used in these studies was prepared using tangential flow filtration, following the established procedure (Palmer et al., 2009). Expired units of human red blood cells were purchased from the Transfusion Services at The Ohio State University's Wexner Medical Center. The concentration of Hb was assessed through spectrophotometric analysis.

### 2.2 Cell culture method

Conditionally immortalized mouse podocytes (MPC5) were procured from ATCC (Manassas, Virginia, USA). As previously outlined (Li et al., 2013), the cells were cultivated in RPMI 1640 medium supplemented with 10% fetal bovine serum and 1% penicillin–streptomycin (Sigma‐Aldrich, MI, USA) within a constant temperature incubator with 5% CO_2_ at 37°C.

### 2.3 Cell grouping procedure

MPC5 cells in the logarithmic growth stage were harvested and categorized as follows:

Blank Group: Cultured in standard RPMI 1640 medium.

Hb Group: Cultured in RPMI 1640 medium containing 5 μM Hemoglobin (comparable to around a 0.3 g/dL plasma Hb in humans).

The cells were cultured in different groups: cell media culture, Hb, ApoHb‐Hp, or Hb + ApoHb‐Hp for 24 h.

### 2.4 Propidium iodide and MTT assay for cell viability

Cell viability was assessed using the 3‐(4,5‐dimethylthiazol‐2‐yl)‐2,5‐diphenyltetrazolium Bromide (MTT) assay. MPC5 cells from different groups were cultured in 96‐well plates (1 × 10^4^ cells/well), incubated with MTT solution (M405849‐1Set, Sigma) at a final concentration of 1 mg/mL for 4 h at 37°C. Formazan crystals were dissolved with 150 μL/well of DMSO, and absorbance at 570 nm was measured with a microplate reader.

Cell death was furthered examined using Hoechst 33342/propidium iodide (PI) double fluorescence staining kits (CA1120, Solarbio). MPC5 cells in the logarithmic growth stage were cultured in 6‐well plates and stained with Hoechst 33342 solution in darkness at 37°C for 10 min, followed by PI staining in darkness at 25°C for 15 min.

### 2.5 Immunofluorescent staining

Cell slides from different groups were prepared and fixed with 4% paraformaldehyde. Subsequently, the slides were washed with PBS, sealed with goat serum at room temperature for 30 min, and incubated with anti‐Nephrin (1:500, ab216341, Abcam) overnight at 4°C. After washing in PBS with 0.05% Tween‐20, slides were incubated with goat anti‐rabbit secondary antibody Alexa Fluor® 594 IgG H&L (2 μg/mL, ab150080) in the absence of light. Nuclei were stained with 4′,6‐diamidino‐2‐phenylindole (DAPI) and photographed under a fluorescence microscope. The nephrin staining was quantified by counting the number of nephrin‐positive cells and divide them by the number of DAPI positive cells.

#### 2.5.1 Measurement of reactive oxygen species

Reactive oxygen species levels in MPC5 cells were measured using the 2′,7′‐dichlorodihydrofluorescein diacetate (DCFH‐DA) (Invitrogen—C6827) fluorescence probe. Kits were utilized for measuring ROS levels in MPC5 cells. Cells from each group were collected, diluted with PBS, seeded into 6‐well plates, and treated with DCFHDA solution with a final concentration of 5 mM at 37°C for 30 min. Fluorescence intensity was detected by a fluorescence microscope, with the emission wavelength set at 530 nm and the excitation wavelength set at 485 nm, following kit instructions.

#### 2.5.2 Image acquisition

Fluorescent imaging measurements were performed using Olympus BX61.

Fluorescence Motorized Polarization DIC (Tokyo, Japan) equipped the appropriate fluorescent filters (Alexa Fluor® 594/DAPI for Nephrin images and DCFH‐DA for ROS) and a UPlanApo 40× magnification lens. Fluorescence was acquired using a high‐light‐sensitive camera (ORCA‐Fusion Digital CMOS camera C14440‐20UP, Hamamatsu Photonics, Japan).

#### 2.5.3 Detection of OS‐related indicators and inflammatory factors

MPC5 cells from each group were collected, and total protein was extracted using radioimmunoprecipitation assay lysate. Protein concentrations were measured using the bicinchoninic acid method. Levels of OS‐related indexes (malondialdehyde, superoxide dismutase, and glutathione) were detected by kits (Invitrogen). Inflammatory cytokine levels (interleukin‐1β and IL‐18) were determined by ELISA kits (Invitrogen). Malondialdehyde (MDA), superoxide dismutase (SOD), and Glutathione (GSH) were quantified using the Elisa assay. Lipid Peroxidation (MDA) Assay Kit (Colorimetric/Fluorometric) (Abcam #118970), Mouse Superoxide Dismutase 1 ELISA Kit (Abcam #285309), and GSH Assay Kit (Colorimetric) (Abcam #239727).

#### 2.5.4 Western blotting (WB)

Total protein was extracted from cells using radioimmunoprecipitation assay lysate (Invitrogen). After measuring protein concentration, proteins (40 μg) were isolated by 10% SDS‐PAGE and transferred onto polyvinylidene fluoride membranes. Membranes were sealed with 5% skim milk for 1 h and incubated with primary antibodies overnight at 4°C. After washing with PBS, membranes were incubated with HRP‐labeled secondary antibodies (Goat anti‐Human IgG Fc Secondary Antibody, HRP—ThemoFisher A18805) and developed by enhanced chemiluminescence (SuperSignal West Pico PLUS Chemiluminescent Substrate—ThermoFisher 34,580). Observations and photographs were made using GAPDH as an internal reference.

#### 2.5.5 Statistical analysis

All experiments were repeated three times. The results are presented as the mean ± the standard deviation. A one‐way ANOVA was performed to compare the means across the four groups. Post hoc comparisons were conducted using Tukey's HSD test to identify specific group differences. A P‐value of less than 0.05 was considered statistically significant. (GraphPad Prism 9, GraphPad Software, Inc., San Diego, CA).

## 3 RESULTS

### 3.1 ApoHb‐hp attenuated MPC5 cell damage induced by hemoglobin

**FIGURE 1:**
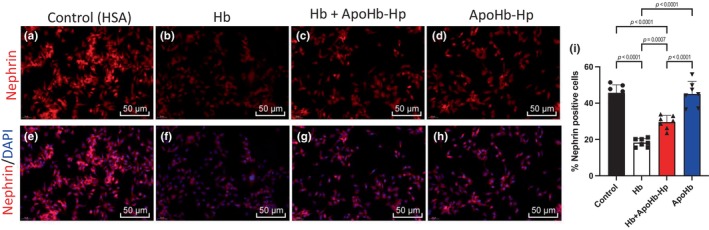
Immunofluorescent staining of Nephrin‐positive podocytes. Podocytes were cultured under various conditions to assess Nephrin expression, an essential protein for the filtration barrier. Reduced Nephrin detection may indicate membrane disruption. (a–d) Representative images of podocytes stained for Nephrin (red) and cultured with: HSA (a), Hb (b), Hb + ApoHb‐Hp (c), and ApoHb‐Hp (d). (e–h) Combined staining for Nephrin (red) and DAPI (blue) under the same conditions: HSA (e), Hb (f), Hb + ApoHb‐Hp (g), and ApoHb‐Hp (h). (i) Quantification of Nephrin‐positive cells across different conditions. Data presented as mean ± SD.

Cells were fluorescently stained with DAPI and Nephrin as shown in Figure 1. Fluorescent quantification of cells was done, resulting in a significantly lower percentage of nephrin‐positive cells in the Hb cultured group compared to the control, ApoHb‐Hp, and Hb + ApoHb‐Hp groups. Hb + ApoHb‐Hp group had significantly higher nephrin levels than that of the Hb group, but they were significantly lower when compared to control and ApoHb‐Hp groups.

**FIGURE 2:**
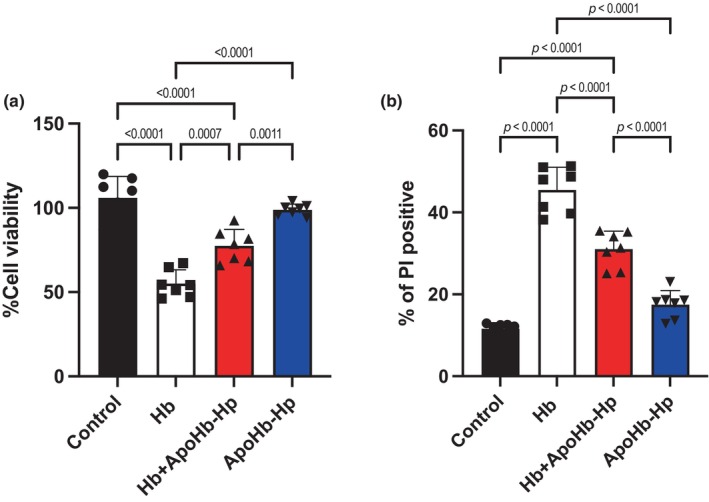
Assessment of MPC5 podocyte viability and Propidium Iodide (PI) staining. (a) Viability of MPC5 podocytes cultured under various conditions, expressed as a percentage. (b) Proportion of PI‐positive MPC5 podocytes indicating cell membrane compromise. Podocytes were treated with: Control (HSA), Hb, Hb + ApoHb‐Hp, and ApoHb‐Hp.

MTT assay to detect cell viability was done and it is shown in Figure 2. The percentage of cell viability was significantly highest in the control and the ApoHb‐Hp groups, compared to Hb and Hb + ApoHb‐Hp groups. Moreover, the Hb group cell viability was significantly lower than all the groups including the Hb + ApoHb‐Hp group suggesting a protective mechanism from the therapeutic against Hb.

**FIGURE 3:**
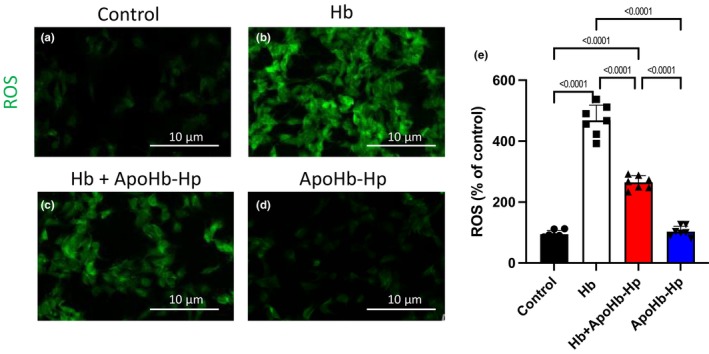
Reactive Oxygen Species (ROS) detection in podocytes through immunofluorescence and quantification. (a–d) Representative images of immunofluorescent staining for ROS (green) in MPC5. Podocytes cultured under different conditions: HSA (a), Hb (b), Hb + ApoHb‐Hp (c), and ApoHb‐Hp (d). (e) Quantification of ROS‐positive cells, expressed as a percentage.

ROS detected with DCFH‐DA fluorescence images with their respective quantification are shown in Figure 3. Hb group had significantly higher ROS production compared to all groups. Hb + ApoHb‐Hp had a significantly lower ROS production compared to Hb; however, it was significantly higher compared to the control and ApoHb‐Hp.

Lastly, for the all the measurements including nephrin‐positive cells, cell viability percentage, and ROS‐positive cells in Figures 1, 2, 3, there was no significant difference between the control and the ApoHb‐Hp group, showing that biocompatibility and safety of the therapeutic.

### 3.2 ApoHb‐hp protects from protein protective mechanisms depletion induced by hemoglobin

**FIGURE 4:**
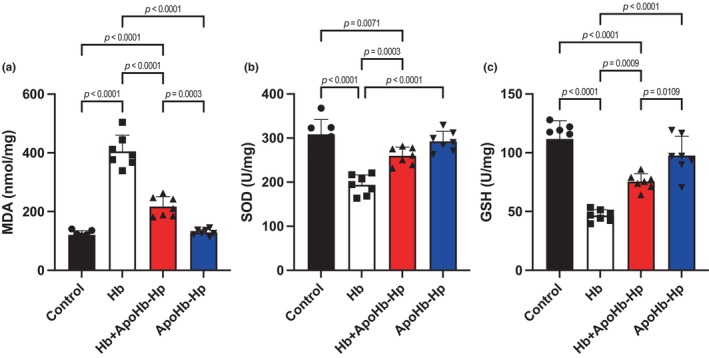
Quantification of Reactive Oxygen Species (ROS) cellular response in podocytes. Podocytes were assessed for cellular responses to oxidative stress under various conditions. The Hb‐treated group demonstrates increased cellular damage, potentially leading to apoptosis. (a) Quantification of Malondialdehyde (MDA) levels, indicating lipid peroxidation. (b) Quantification of Superoxide Dismutase (SOD) activity, a key antioxidant enzyme. (c) Quantification of Glutathione (GSH) levels, a major cellular antioxidant.

ELISA quantification for Malondialdehyde (MDA), superoxide dismutase (SOD), and Glutathione (GSH) concentrations are shown in Figure 4. MDA is significantly higher in the Hb group compared to the other groups. It can be seen how the Hb + ApoHb‐Hp group has a significant decrease compared to the Hb group. However, the ApoHb‐Hp group and the control group do not show significant differences between each other. SOD and GSH are significantly lower in the Hb group compared to all groups. The Hb + ApoHb‐Hp group showed a significant increase compared to Hb showing its protective mechanism. There were no significant changes between the ApoHb‐Hp and control groups.

### 3.3 Activation of NLPR3 inflammasome signaling cascade in the presence of hemoglobin compared to ApoHb‐hp

**FIGURE 5:**
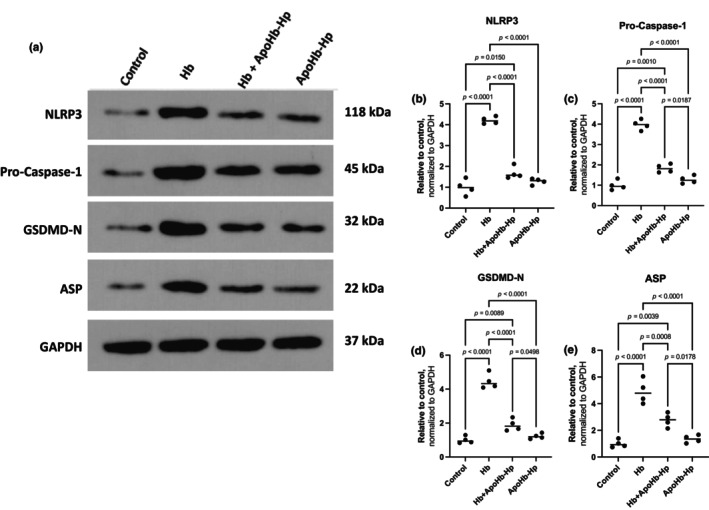
Western blot analysis of protein expression in the NLRP3 inflammasome pathway. (a) Representative Western blot images showing protein expression levels for NLRP3, Pro‐caspase‐1, GSDMD‐N, and ASP in podocytes treated under various conditions. (b–e) Quantification of protein expression levels for NLRP3 (b), Pro‐caspase‐1 (c), GSDMD‐N (d), and ASP (e), with values normalized to GAPDH as the loading control. Increase in expression of the molecules in the pathway is indicative of cell pyropoptosis further down the cascade.

The western blot showcasing the protein levels of NLRP3, Pro‐Caspase‐1, gasdermin D N‐terminal domain (GSDMD‐N), apoptosis‐associated speck‐like protein (ASP), and GAPDH is shown in Figure 5. It can be seen how NLRP3, Pro‐Caspase‐1, GSDMD‐N, and ASP are at a significantly higher expression in the Hb group, compared to the rest of the groups. The ApoHb‐Hp with and without Hb groups have significant lower protein levels than the Hb group, with the control showing the lowest levels of all. The ApoHb‐Hp group did not have significant differences compared to the control, showing equal and minimal NLRP3 pathway activation as the control.

**FIGURE 6:**
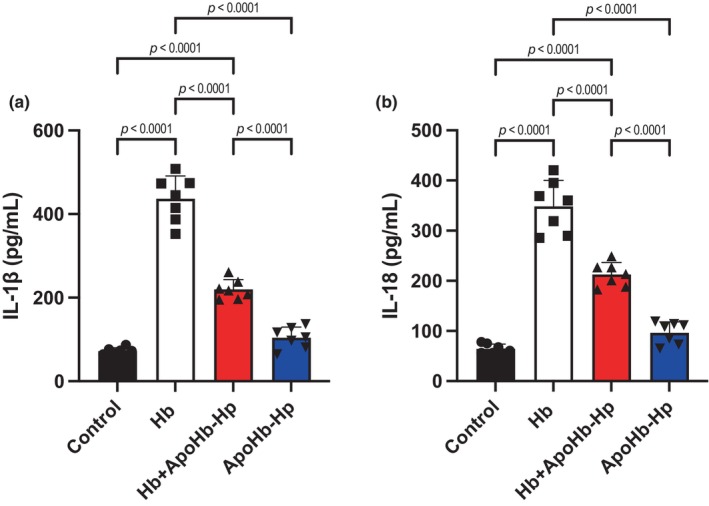
Quantification of inflammatory cytokines IL‐1β and IL‐18 in podocytes. (a) Quantification of interleukin‐1 beta (IL‐1β) levels across different treatment conditions. (b) Quantification of interleukin‐18 (IL‐18) levels across the same conditions.

Lastly, inflammatory cytokines levels for, IL‐1β and IL‐18, are shown in Figure 6. For both inflammatory cytokines, IL‐1β and IL‐18, they were significantly higher in the Hb group. Compared to the Hb group, the treatment with ApoHb‐Hp showed a significant decrease. Lastly, both the control and the ApoHb‐Hp showed similar values with no significance between each other but were significantly lower when compared to the other groups.

It can be seen in these results that the inflammasome pathway gets activated by Hb leading to damage. The NLPR3 activation pathway is a multiprotein cascade responsible for inflammatory signals and innate immunity. It has been shown in past experiments that the pathway gets activated by ROS production in response to mitochondrial damage and dysfunction (Kelley et al., 2019). Our results illustrate this relationship, since the Hb group has the highest ROS production and therefore the highest protein level of NLRP3. NLPR3 throughout secondary signaling cascade molecules, such as ASP, activates Pro‐Caspase‐1, which aid in maturation and activation of inflammatory cytokines, IL‐1β and IL‐18 (Blevins et al., 2022; Kaneko et al., 2019; Martinon & Tschopp, 2007; Zaharie et al., 2023). Pro‐Caspase‐1 is also in charge of cleaving and activating GSDMD‐N, which oversees programmed cell death, pyroptosis (Shi et al., 2015). Moreover, MDA is a marker used to determine oxidative stress but is also involved as signaling molecule to promote cell death (Ayala et al., 2014). Lastly, GSH depletion is also associated with progression of cell death (Franco & Cidlowski, 2012). The activation of these signaling molecules is illustrated in our results as the Hb group shows the highest levels of ASP, Pro‐Caspase‐1, GSDMD‐N, and MDA with a GSH depletion which relate to a lower percentage of cell viability.

## 4 DISCUSSION

Our findings validate the association between Hb‐induced kidney injury and podocyte damage, underscoring the role of circulating Hb in various detrimental clinical outcomes. Moreover, our results illuminate a protective facet of the ApoHb‐Hp complex against Hb‐induced podocyte damage, suggesting a promising avenue for preventing kidney injury when circulating Hb is unavoidable.

The kidneys are particularly vulnerable during hemolysis, as they serve as the primary route for Hb clearance once natural scavenging systems, like Hp and Hpx, become saturated. This depletion of Hp and Hpx leads to several pathophysiological consequences, including increased oxidative stress, inflammation, and tissue damage. In the absence of these protective proteins, Hb dimers are free to extravasate into tissues and create toxic events.

Hb, released during hemolysis, is highly toxic to the kidneys, particularly affecting podocyte function through oxidative damage, inflammation, and disruption of the filtration barrier. Podocytes, the specialized cells responsible for maintaining the integrity of the glomerular filtration barrier, are especially vulnerable to free Hb and heme toxicity. The mechanisms of injury observed in this study and supported by existing literature indicate that hemoglobin increases oxidative stress through ROS generation. Hb rapidly oxidizes, generating free heme and iron, which catalyze ROS formation (Deuel et al., 2016). The oxidative stress leads to significant damage in renal tissues, compromising podocyte function and disrupting the filtration membrane. However, the introduction of ApoHb‐Hp as a therapeutic allows to stabilize the Hb molecule, preventing it from dimerizing and releasing toxic heme. By reducing heme and iron exposure, the ApoHb‐Hp complex limits oxidative damage and toxicity.

As Hb dimerizes, it releases heme. Heme is highly lipophilic which allows it to insert itself into cellular membranes, disrupting the lipid bilayer, causing lipid peroxidation, and impairing membrane integrity (Ayala et al., 2014). In podocytes, this results in damage to structural proteins like nephrin, which is crucial for maintaining the slit diaphragm. The loss of nephrin and other structural proteins results in compromised filtration, leading to proteinuria and glomerular injury (Ruotsalainen et al., 1999; Verma et al., 2018). Therefore, the sustained expression of nephrin further underscores the potential therapeutic benefits of the ApoHb‐Hp complex in mitigating kidney injury and promoting overall renal health. Additionally, free Hb and heme trigger an inflammatory response by activating the innate immune system, releasing pro‐inflammatory cytokines. This inflammatory response leads to podocyte phenotypic changes and apoptosis, further compromising the integrity of the filtration barrier. This is showcased in our study by decreased cell viability in podocytes from the Hb group and an increase in pro‐inflammatory cytokines. 

As Hb continues to break down, the release of free iron accumulates in podocytes, enhancing ROS production (Brissot et al., 2012). This iron toxicity is particularly damaging to podocytes as it affects mitochondrial function, leading to energy depletion and increasing the rate of apoptosis (Baek et al., 2020). Due to the limited regenerative capacity of podocytes, exposure to free Hb and heme results in cytoskeletal disorganization and permanent loss of these cells. Podocyte injury directly leads to proteinuria, the hallmark of glomerular dysfunction.

The ApoHb‐Hp complex exhibits a therapeutic potential in mitigating these harmful effects by stabilizing Hb and preventing it from dimerization and releasing its heme. This allows to reduce oxidative stress, preserves nephrin expression, and maintains the structural integrity of podocytes. While our study presents the protective efficacy of the ApoHb‐Hp complex in vitro, further investigations are necessary to explore its in vivo application.

Hb toxicity can extend beyond podocytes into the renal tubules. Hb's tendency to dimerize and precipitate in the proximal tubules contributes to tubular injury through oxidative stress and inflammation (Belcher et al., 2010; Deuel et al., 2016; Nath et al., 2022). Additionally, the scavenging of NO, an important blood flow regulator, by Hb contributes to vasoconstriction, reducing renal perfusion and worsening overall kidney function (Donadee et al., 2011).

Overall, Hb toxicity arises from multiple mechanisms, including ROS formation, disruption of the filtration membrane, tubular epithelial injury, and NO scavenging. Our findings provide insights into the toxic effects and inflammatory pathways activated by Hb and highlight the protective role of ApoHb‐Hp in modulating these responses. However, it is important to note the limitations of this study, particularly its in vitro nature, which lacks the complexity of in vivo conditions. The experimental setup involved podocytes directly exposed to media containing Hb and ApoHb‐Hp, a condition not reflective of the in vivo scenario. In vivo, conditions encompass endothelial cells, basement membrane cells, and extracellular matrix surrounding podocytes, which limits direct contact with Hb. Future studies should investigate these protective effects in vivo, with a focus on glomerular filtration rate alterations and the interaction between ApoHb‐Hp and native serum proteins such as Hp and Hpx.

In conclusion, this study demonstrates the potential of ApoHb‐Hp as a therapeutic in preventing Hb‐induced renal toxicities, with promising implications for enhancing cell survival and mitigating inflammatory cascades in vitro. Further exploration in animal models and clinical settings will be essential to validate these findings and develop effective therapies for hemolysis‐induced kidney injury.

## FUNDING INFORMATION

This work was supported by National Institutes of Health (NIH) grants R01HL159862, R01HL158076, and R01HL162120 and the National Heart, Lung, and Blood Institute (NHLBI) T3 2 graduate research training grant HL160507.

## ETHICAL STATEMENT

No animal or human studies are involved. Ethical statement is not applicable.

## References

[phy270132-bib-0001] Andersen, C. B. F. , Torvund‐Jensen, M. , Nielsen, M. J. , Oliveira, C. L. P. , Hersleth, H.‐P. , Andersen, N. H. , Pedersen, J. S. , Andersen, G. R. , & Moestrup, S. K. (2012). Structure of the haptoglobin‐haemoglobin complex. Nature, 489, 456–459. 10.1038/nature11369 22922649

[phy270132-bib-0002] Ayala, A. , Muñoz, M. F. , & Argüelles, S. (2014). Lipid peroxidation: Production, metabolism, and signaling mechanisms of malondialdehyde and 4‐Hydroxy‐2‐Nonenal. Oxidative Medicine and Cellular Longevity, 2014, 360438. 10.1155/2014/360438 24999379 PMC4066722

[phy270132-bib-0003] Baek, J. H. , Shin, H. K. H. , Gao, Y. , & Buehler, P. W. (2020). Ferroportin inhibition attenuates plasma iron, oxidant stress, and renal injury following red blood cell transfusion in Guinea pigs. Transfusion (Paris), 60, 513–523. 10.1111/trf.15720 32064619

[phy270132-bib-0004] Baek, J. H. , Yalamanoglu, A. , Brown, R. P. , Saylor, D. M. , Malinauskas, R. A. , & Buehler, P. W. (2018). Renal toxicodynamic effects of extracellular hemoglobin after acute exposure. Toxicological Sciences, 166, 180–191. 10.1093/toxsci/kfy193 30085279

[phy270132-bib-0005] Belcher, J. D. , Beckman, J. D. , Balla, G. , Balla, J. , & Vercellotti, G. (2010). Heme degradation and vascular injury. Antioxidants & Redox Signaling, 12, 233–248. 10.1089/ars.2009.2822 19697995 PMC2821146

[phy270132-bib-0006] Blevins, H. M. , Xu, Y. , Biby, S. , & Zhang, S. (2022). The NLRP3 inflammasome pathway: A review of mechanisms and inhibitors for the treatment of inflammatory diseases. Frontiers in Aging Neuroscience, 14, 879021. 10.3389/fnagi.2022.879021 35754962 PMC9226403

[phy270132-bib-0007] Brissot, P. , Ropert, M. , Le Lan, C. , & Loréal, O. (2012). Non‐transferrin bound iron: A key role in iron overload and iron toxicity. Biochimica et Biophysica Acta (BBA)—General Subjects, 1820, 403–410. 10.1016/j.bbagen.2011.07.014 21855608

[phy270132-bib-0008] Buehler, P. W. , Abraham, B. , Vallelian, F. , Linnemayr, C. , Pereira, C. P. , Cipollo, J. F. , Jia, Y. , Mikolajczyk, M. , Boretti, F. S. , Schoedon, G. , Alayash, A. I. , & Schaer, D. J. (2009). Haptoglobin preserves the CD163 hemoglobin scavenger pathway by shielding hemoglobin from peroxidative modification. Blood, 113, 2578–2586. 10.1182/blood-2008-08-174466 19131549

[phy270132-bib-0009] Buehler, P. W. , & D'Agnillo, F. (2010). Toxicological consequences of extracellular hemoglobin: Biochemical and physiological perspectives. Antioxidants & Redox Signaling, 12, 275–291. 10.1089/ars.2009.2799 19659434

[phy270132-bib-0010] Buehler, P. W. , Humar, R. , & Schaer, D. J. (2020). Haptoglobin therapeutics and compartmentalization of cell‐free hemoglobin toxicity. Trends in Molecular Medicine, 26, 683–697. 10.1016/j.molmed.2020.02.004 32589936

[phy270132-bib-0011] Cabrales, P. , Han, G. , Nacharaju, P. , Friedman, A. J. , & Friedman, J. M. (2011). Reversal of hemoglobin‐induced vasoconstriction with sustained release of nitric oxide. American Journal of Physiology. Heart and Circulatory Physiology, 300, H49–H56. 10.1152/ajpheart.00665.2010 21057038 PMC3023241

[phy270132-bib-0012] Deuel, J. W. , Schaer, C. A. , Boretti, F. S. , Opitz, L. , Garcia‐Rubio, I. , Baek, J. H. , Spahn, D. R. , Buehler, P. W. , & Schaer, D. J. (2016). Hemoglobinuria‐related acute kidney injury is driven by intrarenal oxidative reactions triggering a heme toxicity response. Cell Death & Disease, 7, e2064. 10.1038/cddis.2015.392 26794659 PMC4816175

[phy270132-bib-0013] Deuel, J. W. , Vallelian, F. , Schaer, C. A. , Puglia, M. , Buehler, P. W. , & Schaer, D. J. (2015). Different target specificities of haptoglobin and hemopexin define a sequential protection system against vascular hemoglobin toxicity. Free Radical Biology & Medicine, 89, 931–943. 26475040 10.1016/j.freeradbiomed.2015.09.016

[phy270132-bib-0014] Donadee, C. , Raat, N. J. H. , Kanias, T. , Tejero, J. , Lee, J. S. , Kelley, E. E. , Zhao, X. , Liu, C. , Reynolds, H. , Azarov, I. , Frizzell, S. , Meyer, E. M. , Donnenberg, A. D. , Qu, L. , Triulzi, D. , Kim‐Shapiro, D. B. , & Gladwin, M. T. (2011). Nitric oxide scavenging by red cell microparticles and cell free hemoglobin as a mechanism for the red cell storage lesion. Circulation, 124, 465–476. 10.1161/CIRCULATIONAHA.110.008698 21747051 PMC3891836

[phy270132-bib-0015] Dutra, F. F. , Alves, L. S. , Rodrigues, D. , Fernandez, P. L. , de Oliveira, R. B. , Golenbock, D. T. , Zamboni, D. S. , & Bozza, M. T. (2014). Hemolysis‐induced lethality involves inflammasome activation by heme. Proceedings of the National Academy of Sciences, 111, E4110–E4118. 10.1073/pnas.1405023111 PMC4191786 25225402

[phy270132-bib-0016] Feola, M. , Simoni, J. , Tran, R. , & Canizaro, P. C. (1988). Mechanisms of toxicity of hemoglobin solutions. Biomaterials, Artificial Cells, and Artificial Organs, 16, 217–226. 10.3109/10731198809132571 3179466

[phy270132-bib-0017] Franco, R. , & Cidlowski, J. A. (2012). Glutathione efflux and cell death. Antioxidants & Redox Signaling, 17, 1694–1713. 10.1089/ars.2012.4553 22656858 PMC3474185

[phy270132-bib-0018] Gentinetta, T. , Belcher, J. D. , Brügger‐Verdon, V. , Adam, J. , Ruthsatz, T. , Bain, J. , Schu, D. , Ventrici, L. , Edler, M. , Lioe, H. , Patel, K. , Chen, C. , Nguyen, J. , Abdulla, F. , Zhang, P. , Wassmer, A. , Jain, M. , Mischnik, M. , Pelzing, M. , … Höbarth, G. (2022). Plasma‐derived hemopexin as a candidate therapeutic agent for acute Vaso‐occlusion in sickle cell disease: Preclinical evidence. Journal of Clinical Medicine, 11, 630. 10.3390/jcm11030630 35160081 PMC8836474

[phy270132-bib-0019] Huang, Y.‐X. , Wu, Z.‐J. , Huang, B.‐T. , & Luo, M. (2013). Pathway and mechanism of pH dependent human hemoglobin tetramer‐dimer‐monomer dissociations. PLoS One, 8, e81708. 10.1371/journal.pone.0081708 24312337 PMC3842943

[phy270132-bib-0020] Kaneko, N. , Kurata, M. , Yamamoto, T. , Morikawa, S. , & Masumoto, J. (2019). The role of interleukin‐1 in general pathology. Inflammation and Regeneration, 39, 12. 10.1186/s41232-019-0101-5 31182982 PMC6551897

[phy270132-bib-0021] Kawachi, H. , Miyauchi, N. , Suzuki, K. , Han, G. D. , Orikasa, M. , & Shimizu, F. (2006). Role of podocyte slit diaphragm as a filtration barrier (review article). Nephrology, 11, 274–281. 10.1111/j.1440-1797.2006.00583.x 16889564

[phy270132-bib-0022] Kelley, N. , Jeltema, D. , Duan, Y. , & He, Y. (2019). The NLRP3 inflammasome: An overview of mechanisms of activation and regulation. International Journal of Molecular Sciences, 20, 3328. 10.3390/ijms20133328 31284572 PMC6651423

[phy270132-bib-0023] Kocylowski, M. K. , Aypek, H. , Bildl, W. , Helmstädter, M. , Trachte, P. , Dumoulin, B. , Wittösch, S. , Kühne, L. , Aukschun, U. , Teetzen, C. , Kretz, O. , Gaal, B. , Kulik, A. , Antignac, C. , Mollet, G. , Köttgen, A. , Göcmen, B. , Schwenk, J. , Schulte, U. , … Grahammer, F. (2022). A slit‐diaphragm‐associated protein network for dynamic control of renal filtration. Nature Communications, 13, 6446. 10.1038/s41467-022-33748-1 PMC9616960 36307401

[phy270132-bib-0024] Leger, R. M. , Jain, S. , Nester, T. A. , & Kaplan, H. (2015). Drug‐induced immune hemolytic anemia associated with anti‐carboplatin and the first example of anti‐paclitaxel. Transfusion (Paris), 55, 2949–2954. 10.1111/trf.13255 26264449

[phy270132-bib-0041] Li D , Wang N , Zhang L , Hanyu Z , Xueyuan B , Fu B , Shaoyuan C , Zhang W , Xuefeng S , Li R , Chen X . Mesenchymal stem cells protect podocytes from apoptosis induced by high glucose via secretion of epithelial growth factor. Stem Cell Res Ther. 2013;4:103. 10.1186/scrt314. 24004644 PMC3856604

[phy270132-bib-0025] Martinon, F. , & Tschopp, J. (2007). Inflammatory caspases and inflammasomes: Master switches of inflammation. Cell Death and Differentiation, 14, 10–22. 10.1038/sj.cdd.4402038 16977329

[phy270132-bib-0026] Materne, L. A. , Hunsicker, O. , Menk, M. , & Graw, J. A. (2021). Hemolysis in patients with extracorporeal membrane oxygenation therapy for severe acute respiratory distress syndrome—a systematic review of the literature. International Journal of Medical Sciences, 18, 1730–1738. 10.7150/ijms.50217 33746589 PMC7976579

[phy270132-bib-0027] Muller‐Eberhard, U. , Javid, J. , Liem, H. H. , Hanstein, A. , & Hanna, M. (1968). Brief report: Plasma concentrations of hemopexin, haptoglobin and heme in patients with various hemolytic diseases. Blood, 32, 811–815. 5687939

[phy270132-bib-0028] Munoz, C. J. , Pires, I. S. , Baek, J. H. , Buehler, P. W. , Palmer, A. F. , & Cabrales, P. (2020). Apohemoglobin‐haptoglobin complex attenuates the pathobiology of circulating acellular hemoglobin and heme. The American Journal of Physiology—Heart and Circulatory Physiology, 318, H1296–H1307. 32302494 10.1152/ajpheart.00136.2020 PMC7346542

[phy270132-bib-0029] Nath, K. A. , Singh, R. D. , Croatt, A. J. , & Adams, C. M. (2022). Heme proteins and kidney injury: Beyond rhabdomyolysis. Kidney360, 3, 1969–1979. 10.34067/KID.0005442022 36514409 PMC9717624

[phy270132-bib-0030] Palmer, A. F. , Sun, G. , & Harris, D. R. (2009). Tangential flow filtration of hemoglobin. Biotechnology Progress, 25, 189–199. 10.1002/btpr.119 19224583 PMC2647581

[phy270132-bib-0031] Reiser, J. , & Altintas, M. M. (2016). Podocytes [version 1; peer review: 2 approved]. F1000Research, 5(F1000 Faculty Rev), 114. 10.12688/f1000research.7255.1

[phy270132-bib-0032] Ruotsalainen, V. , Ljungberg, P. , Wartiovaara, J. , Lenkkeri, U. , Kestilä, M. , Jalanko, H. , Holmberg, C. , & Tryggvason, K. (1999). Nephrin is specifically located at the slit diaphragm of glomerular podocytes. Proceedings of the National Academy of Sciences, 96, 7962–7967. 10.1073/pnas.96.14.7962 PMC22170 10393930

[phy270132-bib-0033] Sam, R. , Haghighat, L. , Kjellstrand, C. M. , & Ing, T. S. (2023). Hemolysis during hemodialysis [online]. In Handbook of dialysis therapy (pp. 457–466). ScienceDirect. https://www.ncbi.nlm.nih.gov/pmc/articles/PMC7151848/

[phy270132-bib-0034] Schaer, C. A. , Deuel, J. W. , Schildknecht, D. , Mahmoudi, L. , Garcia‐Rubio, I. , Owczarek, C. , Schauer, S. , Kissner, R. , Banerjee, U. , & Palmer, A. F. (2016). Haptoglobin preserves vascular nitric oxide signaling during hemolysis. American Journal of Respiratory and Critical Care Medicine, 193, 1111–1122. 26694989 10.1164/rccm.201510-2058OC PMC4872667

[phy270132-bib-0035] Sears, D. A. (1970). Disposal of plasma heme in normal man and patients with intravascular hemolysis. The Journal of Clinical Investigation, 49, 5–14. 4188269 10.1172/JCI106222 PMC322438

[phy270132-bib-0036] Shi, J. , Zhao, Y. , Wang, K. , Shi, X. , Wang, Y. , Huang, H. , Zhuang, Y. , Cai, T. , Wang, F. , & Shao, F. (2015). Cleavage of GSDMD by inflammatory caspases determines pyroptotic cell death. Nature, 526, 660–665. 10.1038/nature15514 26375003

[phy270132-bib-0037] Tolosano, E. , & Altruda, F. (2002). Hemopexin: Structure, function, and regulation. DNA and Cell Biology, 21, 297–306. 12042069 10.1089/104454902753759717

[phy270132-bib-0038] Vallelian, F. , Buehler, P. W. , & Schaer, D. J. (2022). Hemolysis, free hemoglobin toxicity, and scavenger protein therapeutics. Blood, 140, 1837–1844. 10.1182/blood.2022015596 35660854 PMC10653008

[phy270132-bib-0039] Verma, R. , Venkatareddy, M. , Kalinowski, A. , Li, T. , Kukla, J. , Mollin, A. , Cara‐Fuentes, G. , Patel, S. R. , & Garg, P. (2018). Nephrin is necessary for podocyte recovery following injury in an adult mature glomerulus. PLoS One, 13, e0198013. 10.1371/journal.pone.0198013 29924795 PMC6010211

[phy270132-bib-0040] Zaharie, R. , Valean, D. , Popa, C. , Fetti, A. , Zdrehus, C. , Puia, A. , Usatiuc, L. , Schlanger, D. , & Zaharie, F. (2023). The multifaceted role and regulation of Nlrp3 inflammasome in colitis‐associated Colo‐rectal cancer: A systematic review. International Journal of Molecular Sciences, 24, 3472. 10.3390/ijms24043472 36834883 PMC9959003

